# Production and characterization of single-chain variable fragment antibodies targeting the breast cancer tumor marker nectin-4

**DOI:** 10.3389/fimmu.2023.1292019

**Published:** 2024-01-15

**Authors:** Ching-Hsuan Liu, Sy-Jye Leu, Chi-Hsin Lee, Cheng-Yuan Lin, Wei-Chu Wang, Bor-Yu Tsai, Yu-Ching Lee, Chi-Long Chen, Yi-Yuan Yang, Liang-Tzung Lin

**Affiliations:** ^1^ Department of Microbiology and Immunology, School of Medicine, College of Medicine, Taipei Medical University, Taipei, Taiwan; ^2^ Department of Microbiology & Immunology, Dalhousie University, Halifax, Nova Scotia, Canada; ^3^ Graduate Institute of Medical Sciences, College of Medicine, Taipei Medical University, Taipei, Taiwan; ^4^ School of Medical Laboratory Science and Biotechnology, College of Medical Science and Technology, Taipei Medical University, Taipei, Taiwan; ^5^ Ph.D. Program in Medical Biotechnology, College of Medical Science and Technology, Taipei Medical University, Taipei, Taiwan; ^6^ Navi Bio-Therapeutics Inc., Taipei, Taiwan; ^7^ The Center of Translational Medicine, Taipei Medical University, Taipei, Taiwan; ^8^ Department of Pathology, School of Medicine, College of Medicine, Taipei Medical University and Taipei Medical University Hospital, Taipei, Taiwan; ^9^ Core Laboratory of Antibody Generation and Research, Taipei Medical University, Taipei, Taiwan

**Keywords:** phage display, single-chain variable fragment antibody, nectin-4, tumor marker, breast cancer

## Abstract

**Background:**

Nectin-4 is a novel biomarker overexpressed in various types of cancer, including breast cancer, in which it has been associated with poor prognosis. Current literature suggests that nectin-4 has a role in cancer progression and may have prognostic and therapeutic implications. The present study aims to produce nectin-4-specific single-chain variable fragment (scFv) antibodies and evaluate their applications in breast cancer cell lines and clinical specimens.

**Methods:**

We generated recombinant nectin-4 ectodomain fragments as immunogens to immunize chickens and the chickens' immunoglobulin genes were amplified for construction of anti-nectin-4 scFv libraries using phage display. The binding capacities of the selected clones were evaluated with the recombinant nectin-4 fragments, breast cancer cell lines, and paraffin-embedded tissue sections using various laboratory approaches. The binding affinity and *in silico* docking profile were also characterized.

**Results:**

We have selected two clones (S21 and L4) from the libraries with superior binding capacity. S21 yielded higher signals when used as the primry antibody for western blot analysis and flow cytometry, whereas clone L4 generated cleaner and stronger signals in immunofluorescence and immunohistochemistry staining. In addition, both scFvs could diminish attachment-free cell aggregation of nectin-4-positive breast cancer cells. As results from ELISA indicated that L4 bound more efficiently to fixed nectin-4 ectodomain, molecular docking analysis was further performed and demonstrated that L4 possesses multiple polar contacts with nectin-4 and diversity in interacting residues.

**Conclusion:**

Overall, the nectin-4-specific scFvs could recognize nectin-4 expressed by breast cancer cells and have the merit of being further explored for potential diagnostic and therapeutic applications.

## 1 Introduction

The recombinant antibody molecule single-chain variable fragment (scFv) has emerged as a compelling variant of intact monoclonal antibodies (mAb) due to its reduced molecular size and lower production cost. scFv is an engineered antibody fragment comprising the heavy and light chains' variable domains (V_H_ and V_L_) joined by a short flexible peptide linker. It retains complete monovalent targeting affinity and specificity (1), rendering it theoretically viable for all bench and bedside applications currently relying on intact mAb. Moreover, as a minimized antibody lacking the fragment crystallizable (Fc) domain, scFv exhibits superior pharmacokinetic properties, notably enhanced tumor penetration and low retention rates in non-target organs (2, 3). Furthermore, with regard to production, there is relative ease in and preference for constructing scFv due to the advancement in genetic engineering and phage display technology (3), an *in vitro* method that can produce highly diverse libraries for high-affinity antibody selection (4). scFv can also be efficiently and economically produced in bacteria expression systems since they do not require glycosylation (5). Finally, scFv has excellent potential to be modified and developed into diverse immunoconjugates with varied and enhanced functionality for clinical and laboratory uses (3). As a promising alternative to intact mAb, scFv variants have entered clinical development, representing about 40% of clinically evaluated antibody fragments (6), with cancer being the top target of patented scFvs (3).

Nectin-4, also known as poliovirus receptor-related 4 (PVRL4), is an immunoglobulin (Ig) superfamily member of the nectin family which regulates the formation of cell-cell junctions (7). This adhesion junction protein has three Ig-like domains in its extracellular portion, including one variable (V) type domain and two constant (C) type domains. In contrast to the other members of the nectin family, nectin-4 is highly expressed in the placenta (hence also an embryonic protein) but modestly expressed in the trachea and skin and is absent in most normal human tissues (8). Recently, nectin-4 has been identified as a tumor marker in several types of carcinoma, including lung (9), breast (10), ovarian (11), esophageal (12), gastric (13), pancreatic (14), liver (15), colon (16), and bladder (17) cancers, and has been suggested to promote carcinogenesis (18–22). The upregulation of nectin-4 was first reported in breast cancer, especially in ductal carcinomas, and positively correlated with basal-like markers, which often implies poor prognosis (10). This observation was further supported by a bigger dataset where nectin-4-high triple-negative breast cancer patients had shorter metastasis-free survival (23). Nectin-4 expression is also related to shorter disease-free survival and relapse-free survival in luminal A (24) and luminal B human epidermal growth factor receptor 2 (HER2)-negative (25) breast cancers, suggesting that nectin-4 could be a potential prognostic marker and a therapeutic target of breast cancer.

Given the high level of expression and importance of nectin-4 in cancers, including breast cancer, and the benefits of scFv as an emerging diagnostic and therapeutic tool, we produced anti-nectin-4 scFvs using phage display and characterized their use for detecting nectin-4 in breast cancer cell lines and tissue sections. Their impact on breast cancer cells were also evaluated.

## 2 Materials and methods

### 2.1 Construction and purification of nectin-4 protein fragments

Two recombinant protein fragments, r342p and r864p, were constructed based on the extracellular region of human nectin-4 (accession number: NM_030916). Fragment r342p contained only the membrane-distal V-type domain, while r864p contained all three Ig-like domains. The nucleotide sequence of r342p and r864p were amplified from vectors containing nectin-4 (26) and cloned into the pET-21a vector. The plasmids were amplified in *Escherichia coli* (*E. coli*) with broth containing 50 μl/ml ampicillin and induced by 1 mM isopropyl β-D-1-thiogalactopyranoside (IPTG) at 37°C overnight for protein expression. Pellets were then collected and resuspended in histidine (His) binding buffer containing 6 M urea. The cell membrane was disrupted by sonication and precipitated to release the proteins. Recombinant nectin-4 fragments were purified from the supernatants using Ni Sepharose High Performance (GE Healthcare Life Science, Pittsburgh, PA, USA) according to the manufacturer’s instructions. The purified nectin-4 fragments were further analyzed with sodium dodecyl sulfate-polyacrylamide gel electrophoresis (SDS-PAGE) and western blot.

### 2.2 Immunization and purification of chicken polyclonal IgY

The experimental protocol for chicken immunization was approved by the Institutional Animal Care and Use Committee of Taipei Medical University (TMU). Purified nectin-4 fragments r342p or r864p were dissolved in phosphate buffered saline (PBS) and mixed with complete (for the first immunization) or incomplete Freund’s adjuvant (Sigma-Aldrich, St. Louis, MO, USA). The solutions were then intramuscularly injected into female Leghorn (*Gallus domesticus*) hens for four (r864p) or five (r342p) dosages at the interval of 7 days as previously described (27). Eggs were collected before and after each immunization. Polyclonal IgY were then purified from the egg yolks using the previously reported dextran sulfate method (28) and analyzed for their binding capacity to the recombinant nectin-4 fragments using western blot and enzyme-linked immunosorbent assay (ELISA).

### 2.3 Construction of scFv libraries

Monoclonal scFv antibodies were generated using the previously described phage display method (29, 30) with a few modifications. To establish the cDNA libraries, the immunized hens were sacrificed after the final immunization, and total RNA was extracted from the spleens using Trizol Reagent (Invitrogen, Carlsbad, CA, USA) following the manufacturer’s instructions. After reverse transcription-PCR (RT-PCR), the synthesized cDNA was used to amplify the variable regions of light chains (V_L_) and heavy chains (V_H_) of chicken immunoglobulin genes with the primers (**Supplementary Table S1**): CSCVHo-F and CSCG-B were used to amplify V_H_ with a short linker (GQSSRSS), CSCVHo-FL and CSCG-B were used to amplify V_H_ with a long linker (GQSSRSSGGGGSSGGGGS), and CSCVK and CKJo-B were used to amplify V_L_. This would generate a short linker library and a long linker library for each immunogen. The purified V_H_ and V_L_ DNA fragments were then pooled and further amplified with CSC-F and CSC-B primers to generate full-length scFv genes. These full-length scFv genes were cloned into a pComb3X vector with SfiI (New England Biolabs, Ipswich, MA, USA) to generate constructs that encoded a 6x His tag and a HA tag in their C terminus. The purified plasmids were electroporated into *E. coli*, and the transformed bacteria were then infected with M13 helper phages. Recombinant phages in the supernatant were collected by precipitation with 4% polyethylene glycol 8000 (PEG-8000; Sigma-Aldrich) and 3% NaCl (Merck, Darmstadt, Germany) and resuspended in PBS.

To isolate and amplify the phage-displayed scFv libraries with high specificity, the biopanning steps were carried out using a similar method as previously described (27). Four rounds of biopanning were performed to selectively amplify the phages that displayed nectin-4-specific scFv antibodies. After the fourth round of biopanning, total DNA from the amplified phages was purified and used to transform the heat-shock competent TOP10F’ *E. coli*. Colonies were picked and amplified, after which 0.5 mM IPTG was added to induce scFv expression. Bacterial cultures were then collected, resuspended in His-binding buffer (20 mM sodium phosphate, 0.5 M NaCl, 20 mM imidazole, pH 7.4), and lysed by sonication to release the His-tagged scFvs, which were purified using Ni^2+^ Sepharose columns as previously described (27). The V_L_ and V_H_ genes of the scFv clones were sequenced by Genomics (Taipei, Taiwan) using the OmpA primers. Amino acid sequences of the clones were then determined and aligned to those of the chicken immunoglobulin germline using the BioEdit alignment program.

### 2.4 Cell culture

MCF-7, MDA-MB-231, BT-474, MDA-MB-453, and Vero cells were acquired from the American Type Culture Collection (ATCC; Manassas, VA, USA). All cells were maintained in Dulbecco’s Modified Eagle Medium (Gibco, Thermo Fisher Scientific, Waltham, CA, USA) containing 10% fetal bovine serum (FBS; Gibco), 10 µg/ml of gentamicin (Gibco) and 0.5 µg/ml of Amphotericin B (Gibco). Vero cell overexpressing human nectin-4 (Vero-hNectin-4) was generated using a retroviral transduction method and cultured in the above medium containing additional 1 mg/ml of G418 (InvivoGen, San Diego, CA, USA).

### 2.5 Western blot analysis

Purified proteins or whole cell lysates were analyzed using standard western blot analysis. Briefly, samples were separated by SDS-PAGE, and the gel was then stained with Coomassie blue for protein visualization or transferred to a polyvinylidene fluoride (PVDF) membrane for blocking and antibody incubations. For the detection of recombinant nectin-4, the membrane was incubated with mouse anti-His IgG (1:3000; Bioman Scientific, New Taipei City, Taiwan) and secondary horseradish peroxidase (HRP)-conjugated rabbit anti-mouse IgG (1:5000; Jackson ImmunoResearch, West Grove, PA, USA). The membrane was then visualization by 3, 3’-diaminobenzidine tetrahydrochloride (DAB) staining. For the detection of endogenous nectin-4 from whole cell lysates using scFv, the membrane was incubated with scFv (10 µg/ml), mouse anti-HA secondary antibody (1:5000; Cat# 66006-1, Proteintech, Rosemont, IL, USA), and HRP-conjugated anti-mouse tertiary antibody (1:5000; Cat# 7076, Cell Signaling Technology, Danvers, Massachusetts, USA). Finally, the membrane was stained with Clarity Western ECL Substrate (Bio-Rad) and visualized by ImageQuant™ LAS 4000 (GE Healthcare Life Science).

### 2.6 Indirect ELISA

For the indirect ELISA, 96-well half-area plates were coated with either recombinant nectin-4 fragment or BSA (0.25 µg per well) and blocked with 5% skim milk. To determine the binding capacity of IgY, the coated wells were incubated with serially diluted chicken IgY and incubated with HRP-conjugated donkey anti-chicken IgY (1:5000; Jackson ImmunoResearch). To determine the expression of nectin-4-specific scFvs on the phages after biopanning, 2x diluted phages were added to the wells and further incubated with HRP-conjugated mouse anti-M13 phage antibody (1:3000; GE Healthcare Life Science). To determine the binding capacity of scFvs, the coated wells were incubated with serially diluted scFv primary antibody, goat anti-chicken light chain secondary antibody (1:3000; Bethyl, Montgomery, TX, USA), and the tertiary HRP-conjugated donkey anti-goat IgG (1:5000). All incubations were carried out at 37°C for 1 h, and washing steps with PBST were included between all incubations. After the final incubation, the wells were washed and stained with 3,3’,5,5’-tetramethylbenzidine (TMB; Sigma), and the reaction was stopped by 1 N HCl before the absorbance was read at 450 nm using a Synergy HT plate reader (BioTek, Winooski, VT, USA).

### 2.7 Competitive ELISA

Free nectin-4 fragment r864p was serially diluted and mixed with S21 (20 μg/ml, approximately 666.67 nM) or L4 (1 μg/ml, approximately 33.33 nM) and incubated at 25°C for 1 h, before the mixture was added to plates coated with r864p and incubated at 37°C for 1 h. The blocking, washing, staining, and detection steps were performed using the abovementioned methods. The dissociation constant KD is approximately equal to the concentration of free antigen when the half-maximal ELISA signal is acquired (31). KD values were calculated with variable slope nonlinear regression analysis using GraphPad 9.

### 2.8 Cell-based ELISA

Cells seeded in 24-well plates (2 x 10^5^ cells/well) were washed and fixed with 4% paraformaldehyde (PFA; Affymetrix, Santa Clara, CA, USA) before incubation in 3% BSA in PBS blocking buffer for 1 h at 37°C. The cells were then incubated with various concentrations of scFvs and goat anti-chicken light chain secondary antibody (1:5000). Subsequently, the cells were washed and further incubated with the tertiary HRP-conjugated donkey anti-goat IgG (1:10000). All incubations were carried out at 37°C for 1 h. After the final incubation, the cells were washed and stained with TMB as described above.

### 2.9 Flow cytometry

Cells (5 x 10^4^ cells/sample) were fixed with 10% ethanol and blocked in 3% FBS in PBS blocking buffer. For detection of cell surface nectin-4 using scFv, cells were incubated with scFv primary antibody (37.5 μg/ml), goat anti-chicken light chain secondary antibody (1:400), and rabbit anti-goat IgG Fluor 488-labeled tertiary antibody (1:400; AnaSpec, Fremont, CA, USA). Staining with the commercial PE-conjugated anti-Nectin-4 antibody (FAB2659P, R&D Systems) and its isotype control (IC0041P, R&D Systems) were performed following the manufacturer's instructions and included for comparison. Data were acquired with the BD FACSCalibur Cell Analyzer (BD Biosciences, San Jose, CA, USA).

### 2.10 Immunofluorescence staining

Cells seeded in 96-well plates (2 x 10^4^ cells/well) were washed and fixed with 4% PFA for 10 min at room temperature, then incubated in 3% BSA in PBS blocking buffer for 1 h at room temperature. After which, cells were incubated with scFv primary antibody (0.1 μg/ml), mouse anti-HA secondary antibody (1:500), and goat anti-mouse Alexa Fluor 488 (1:300; Thermo Fisher Scientific) tertiary antibody. All antibody dilutions were prepared in 3% BSA blocking buffer. Finally, the cells were stained with Hoechst nuclear stain (1:500; Sigma-Aldrich) and examined using Invitrogen EVOS™ FL Cell Imaging System (Thermo Fisher Scientific).

### 2.11 Immunohistochemistry staining

Paraffin sections of breast ductal carcinomas and adjacent non-tumor tissues were obtained from Taipei Medical University Joint Biobank as approved by the TMU-Joint Institutional Review Board. Informed consent was waived. Before staining, sections were deparaffinized with xylene and ethanol, and antigen retrieval was performed using the heat-induced method at 121°C for 10 min. Endogenous HRP was inactivated by treating the sections with 3% hydrogen peroxide for 5 min. The sections were then blocked with Background Sniper (Biocare Medical, Pacheco, CA, USA) for 15 min at room temperature. For scFv staining, the slides were incubated with scFv primary antibody (10 μg/ml), mouse anti-HA secondary antibody (1 μg/ml), and the Starr Trek Universal HRP Detection System (Biocare Medical, Pacheco, CA, USA), and counter-stained with hematoxylin. For the commercial anti-nectin-4 antibody staining, the slides were incubated with a polyclonal rabbit anti-nectin-4 antibody (1:600; Cat# HPA010775, Sigma-Aldrich) and visualized with the Starr Trek Universal HRP Detection System and hematoxylin as described above.

### 2.12 Clustering assay

Self-clustering of breast cancer cells was analyzed as previously reported (18). Cells were first detached with enzyme-free cell dissociation buffer (Thermo Fisher Scientific) and resuspended in complete medium. Then 1 x 10^5^ cells were transferred to an Eppendorf tube and incubated in 1 ml complete medium with or without scFv (10 μg/ml) at room temperature. After 1 h, cells were poured into 6-well plates and visualized using Invitrogen EVOS™ FL Cell Imaging System for counting. A total of 5 random fields were analyzed for each well, and clusters with over 5 cells were counted.

### 2.13 Protein-protein docking analysis

The homology model of the scFv L4 was created using SWISS-MODEL (Swiss Institute of Bioinformatics; Basel, Switzerland) based on a scFv template (PDBID: 5VF6) (32). Protein-protein docking was performed using ClusPro 2.0’s antibody docking mode (33). The nectin-4 crystal structure was obtained from the PDB database (PDBID: 4FRW) and used as the ligand molecule, whereas the scFv homology model was used as the receptor molecule. All models were analyzed using the PyMOL Molecular Graphics System (Version 1.7.4, Schrödinger, LLC; Portland, OR, USA) (34).

## 3 Results

### 3.1 Generation of nectin-4-specific polyclonal IgY from immunized chickens

**Figure 1:**
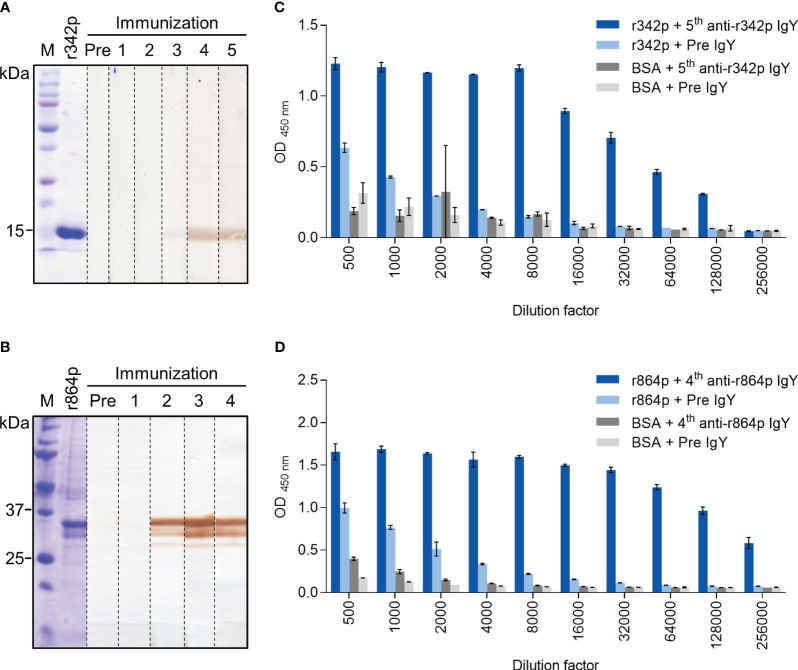
Binding analyses of polyclonal IgY against recombinant nectin-4 fragments r342p and r864p. Hens were immunized with nectin-4 fragments r342p or r864p for 5 or 4 cycles, respectively, and polyclonal IgY was purified from the egg yolks after each immunization cycle. **(A, B)** The r342p and r864p fragments were visualized on SDS-PAGE gel with Coomassie blue staining or transferred to PVDF membrane and immunoblotted with the IgY collected before (“Pre”) or after each immunization cycle. The molecular weight marker (M) is shown on the left. Representative data are shown. **(C, D)** Pre-immunization IgY (“Pre IgY”) or IgY collected after the last immunization cycle (“5^th^ anti-r342p IgY” or “4^th^ anti-r864p IgY”) were serially diluted and evaluated by immunogen- or BSA-coated plate-based indirect ELISA. BSA served as a negative control antigen (mean ± SD, N=2).

Like the other members in the nectin family, nectin-4 has three Ig-like domains in its extracellular portion, including one V-type and two C-type domains (8). To generate polyclonal antibodies against nectin-4, we immunized chickens with recombinant nectin-4 fragments r342p (V domain) and r864p (V-C-C domains). The nectin-4 fragments, after SDS-PAGE and Coomassie blue staining, appeared at the positions of approximately 15 kDa (r342p; **Figure 1A**) and 35 kDa (r864p; **Figure 1B**), respectively. Polyclonal IgY antibodies purified from the immunized chickens were used as primary antibodies to detect these protein fragments on western blots. As shown in **Figure 1A**, the presence of anti-r342p antibodies became prominent after 4 cycles of immunization. On the other hand, anti-r864p antibodies were generated after 2 cycles of immunization (**Figure 1B**). The binding specificity of these antibodies was further evaluated with ELISA. As shown in **Figures 1C, D**, the anti-r342p and anti-r864p antibodies were specific and bound robustly to their immunogens (O.D. > 1.0 for anti-r342p IgY, and O.D. > 1.5 for anti-r864p IgY) with minimal reactivity to BSA. In contrast, the pre-immunization IgY did not show specific binding to the nectin-4 fragments or BSA. These results indicate that we successfully generated nectin-4-specific polyclonal IgY, which were used for the following construction of scFv phage libraries.

### 3.2 ScFv clones in the anti-r342p long linker and anti-r864p short linker phage libraries demonstrate the best nectin-4-binding ability

**Figure 2:**
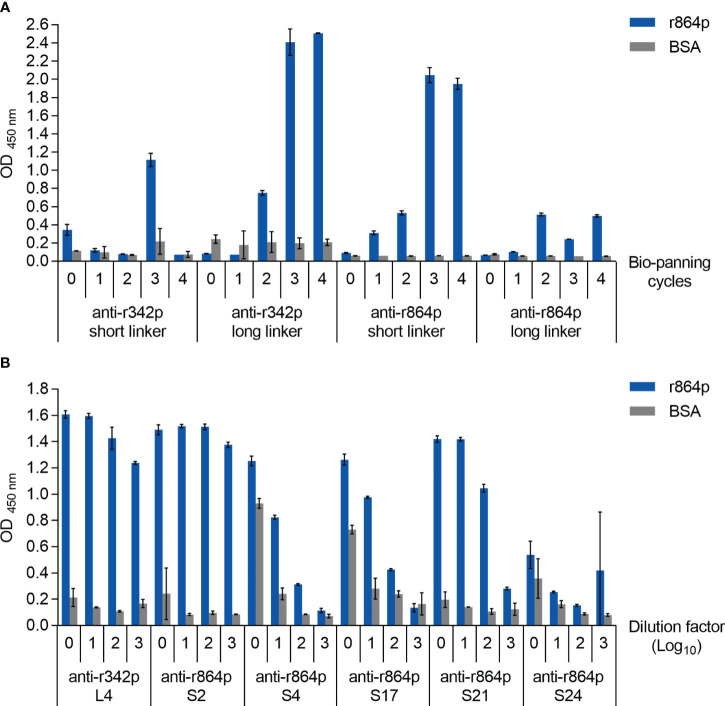
Construction of anti-nectin-4 scFv libraries. **(A)** Anti-r342p and anti-r864p phages from each biopanning cycle were diluted 10x and evaluated for their binding capacity to r864p using plate-based indirect ELISA. BSA served as a negative control antigen (mean ± SD, N=2). **(B)** Purified scFvs from the representative clones were serially diluted (starting concentration: 10 μg/ml) and evaluated for their binding capacity by r864p-coated plate-based indirect ELISA. BSA served as a negative control antigen (mean ± SD, N=2).

We next attempted to produce anti-nectin-4 scFv antibodies using the phage display method. Four scFv phage libraries were generated based on the amplified chicken antibody sequences, namely the anti-r342p short linker library, anti-r342p long linker library, anti-r864p short linker library, and anti-r864p long linker library. These four libraries were subjected to four rounds of biopanning to amplify the phages that expressed nectin-4-specific scFv antibodies. Phages collected before and after each round of biopanning were then analyzed with ELISA for their binding capacity to r864p, the recombinant nectin-4 fragment containing all three extracellular Ig-like domains. As shown in **Figure 2A**, after 3 rounds of biopanning, the binding capacity of the anti-r342p long linker and anti-r864p short linker libraries substantially increased. In contrast, the anti-r342p short linker and anti-r864p long linker libraries had low binding activity to the nectin-4 fragment. None of the libraries reacted to BSA.

To express the scFv antibodies from the anti-r342p long linker and anti-r864p short linker libraries, total DNA from the phages were extracted after the fourth-round biopanning and used to transform TOP10F’ *E. coli* cells. Thirteen and 26 colonies were randomly picked from the anti-r342p long linker and anti-r864p short linker libraries, respectively, for protein purification. Seven colonies from the anti-r342p long (L) linker library and 12 colonies from the anti-r864p short (S) linker library were then selected for sequence analysis based on their specific binding to nectin-4. The V_L_ and V_H_ amino acid sequences of each clone were aligned to those of the chicken immunoglobulin germline. Based on the alignment results, the mutations were mainly found in the complementarity-determining regions (CDR), which generate the paratope for antigen binding. The 7 colonies from the anti-r342p long linker library shared identical amino acid sequences (representative clone: L4), and the 12 colonies from the anti-r864p short linker library resulted in 5 different sequences (representative clones: S2, S4, S17, S21, and S24). After assessing the binding capacity of the representative clones as primary antibodies using ELISA (**Figure 2B**), clones L4 and S21 were selected for subsequent characterization due to their superior activity of recognizing nectin-4 fragment r864p. Clone S2 was not further pursued due to its lower production yield.

### 3.3 Clones L4 and S21 successfully recognize nectin-4 expressed on breast cancer cells

**Figure 3:**
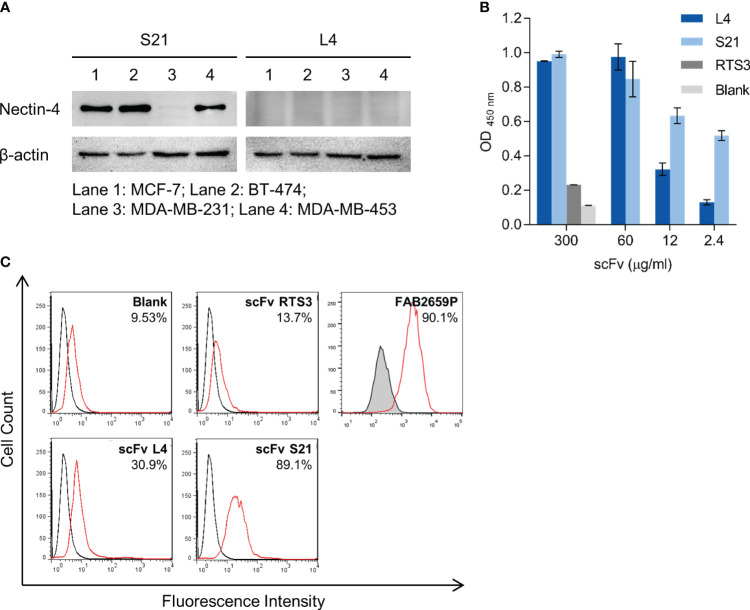
*In vitro* binding analyses of the anti-nectin-4 scFvs S21 and L4. **(A)** The scFvs (10 μg/ml) were used as primary antibodies to detect endogenous nectin-4 in MCF-7, BT-474, and MDA-MB-453 cells in western blot. MDA-MB-231 served as a negative control. Representative data are shown. **(B)** The scFvs were used as primary antibodies to stain MCF-7 cells in cell-based ELISA (mean ± SD, N=2). RTS3 (an anti-snake venom scFv) served as an unrelated control. A control with only the secondary and tertiary antibodies (‘Blank’) was included. **(C)** The scFvs were used as primary antibodies to stain MCF-7 cells in flow cytometry (37.5 μg/ml). Black and red solid lines indicate unstained and stained samples. The blank and RTS3 controls were also included. The commercial PE-conjugated anti-nectin-4 antibody (FAB2659P, R&D Systems; red solid line) and its isotype control (IC0041P, R&D Systems; tinted with black line) were included for comparison. Representative data are shown.

**Figure 4:**
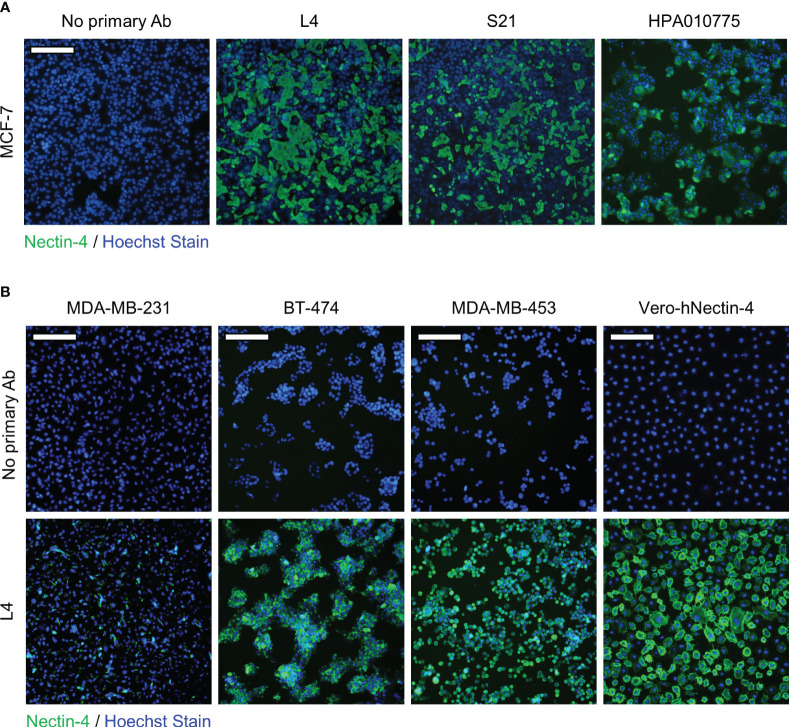
Immunofluorescence staining of breast cancer cell lines with anti-nectin-4 scFv clones. Clone L4 or S21 was used as the primary antibody (0.1 μg/ml) to detect endogenous nectin-4 in **(A)** MCF-7, **(B)** BT474, and MDA-MB-453 cells. Scale bar = 200 μm. Representative data are shown. MDA-MB-231 served as a negative control, and Vero-hNectin-4 served as a positive control. A control with only the secondary and tertiary antibodies (‘No primary antibody’) was included. Staining with the commercial anti-nectin-4 antibody (HPA010775; Sigma-Aldrich) was included for comparison.

To evaluate the application of L4 and S21, we next examined whether these scFvs could detect endogenous nectin-4 expressed in human breast cancer cell lines, including MCF-7 (luminal type A), BT-474 (luminal type B HER2-positive), and MDA-MB-453 (triple negative) (10, 26, 35). The nectin-4-negative MDA-MB-231 breast cancer cells served as a negative control. When used in western blotting as primary antibodies, S21, but not L4, successfully detected nectin-4 in the whole cell lysates at approximately 60 kDa (**Figure 3A**). Both clones could recognize the endogenous nectin-4 in MCF-7 cells using cell-based ELISA, with S21 generating higher signals than L4 after serial dilutions (**Figure 3B**). In flow cytometry analysis, S21 also produced a comparable staining pattern to the commercial PE-conjugated anti-nectin-4 antibody FAB2659P (**Figure 3C**). When used for immunofluorescence surface staining, both L4 and S21 could stain nectin-4 on MCF-7 cells (**Figure 4A**). Notably, L4 produced little background in the nectin-4-negative MDA-MB-231 breast cancer cell (**Figure 4B**), whereas S21 generated higher background (data not shown). L4 could also stain the other nectin-4-positive breast cancer cell lines (including BT-474 and MDA-MB-453) and Vero-hNectin-4 cells. These results suggest that the two clones, S21 and L4, could have different research applications, with L4 having the additional advantage for cell staining.

### 3.4 Immunohistochemistry staining of paraffin-embedded breast cancer tissue sections using scFv L4

**Figure 5:**
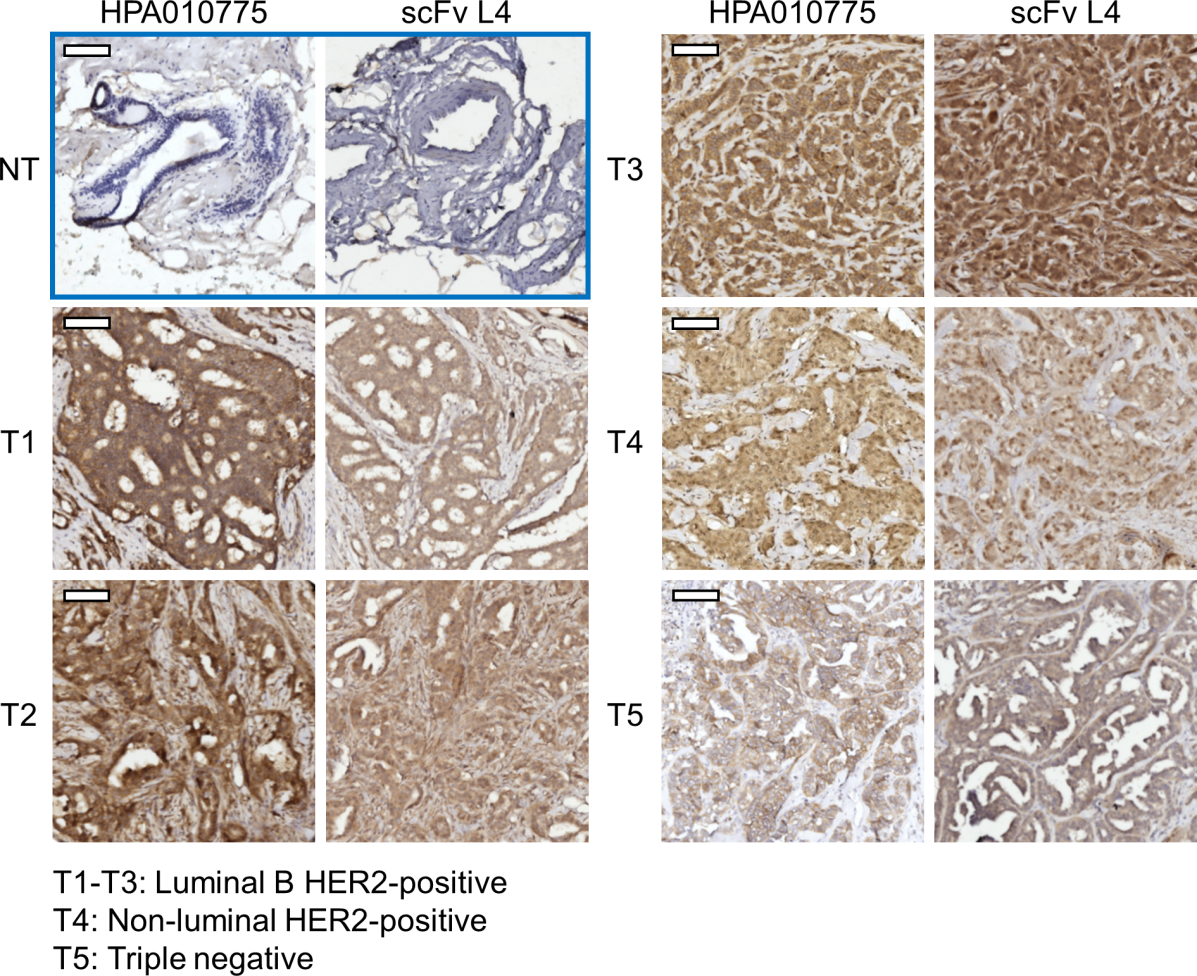
Anti-nectin-4 scFv L4 staining of breast cancer clinical specimens. ScFv L4 was used as the primary antibody (10 μg/ml) for immunohistochemistry staining of breast ductal carcinoma paraffin-embedded tissue sections (T1-T5 from five different patients) and compared with the commercial anti-nectin-4 antibody HPA010775. Non-tumor (NT) tissues were included as a negative control. Scale bar = 100 μm.

To validate the feasibility of using the nectin-4-specific scFv L4 on clinical samples as a diagnostic tool, we further performed immunohistochemistry (IHC) staining of breast ductal carcinoma paraffin-embedded tissue sections with the scFv. As shown in **Figure 5**, L4 yielded comparable staining results to the commercial anti-nectin-4 antibody HPA010775 (Sigma-Aldrich), with minimal background on the non-tumor tissues (NT) and strong signals on the tumor tissues (T1-T5) of different molecular subtypes. This suggests the high sensitivity of scFv L4 binding to native nectin-4 molecule, which supports its potential to be further developed as a tumor-marker-specific diagnostic and/or therapeutic agent.

### 3.5 Assessment of the scFvs’ anti-breast cancer effect *in vitro*

**Figure 6:**
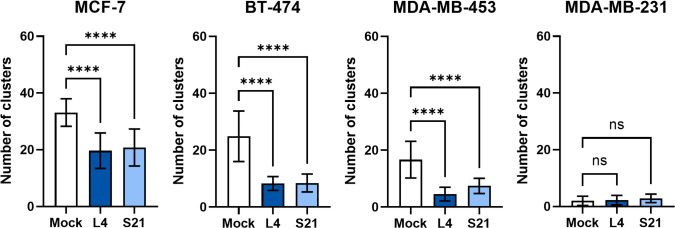
Self-clustering of breast cancer cells with or without anti-nectin-4 scFvs. Nectin-4-positive cells MCF-7, BT474, and MDA-MB-453 were dissociated and allowed to aggregate in medium with or without scFv (10 μg/ml). Cells were then poured into 6-well plates and visualized for counting. Five random fields in the wells were counted for clusters (more than 5 cells) using bright-field microscopy (4X objective lens magnification). Nectin-4-negative MDA-MB-231 cells served as a negative control. Data presented are mean ± SD (N=3). One-way ANOVA with Dunnett’s multiple comparisons test was performed to determine the difference between Mock and scFv treatment groups of each cell line. (****p ≤ 0.0001; ns, not significant).

Given that nectin-4 plays a vital role in the carcinogenesis of breast cancer, we further explored whether the scFvs could inhibit cell growth *in vitro*. Our initial results indicated that the scFvs are not significantly cytotoxic to nectin-4-positive and nectin-4-negative breast cancer cell monolayers (**Supplementary Figure S1**). Nonetheless, considering nectin-4’s contribution to cell-to-cell attachment and tumor cells’ anchorage-independent growth (18), we then performed a clustering assay to evaluate whether the scFvs could inhibit breast cancer cell aggregation, which is important for tumor formation (18). As shown in **Figure 6**, nectin-4-positive breast cancer cells easily formed cell clusters in suspension. More importantly, such self-clustering phenomenon was decreased by the treatment of both scFvs (**Figure 6**), indicating their ability to inhibit nectin-4-positive tumor cell aggregation.

### 3.6 Characterizing the interaction between scFvs and the ectodomain of nectin-4

**Figure 7:**
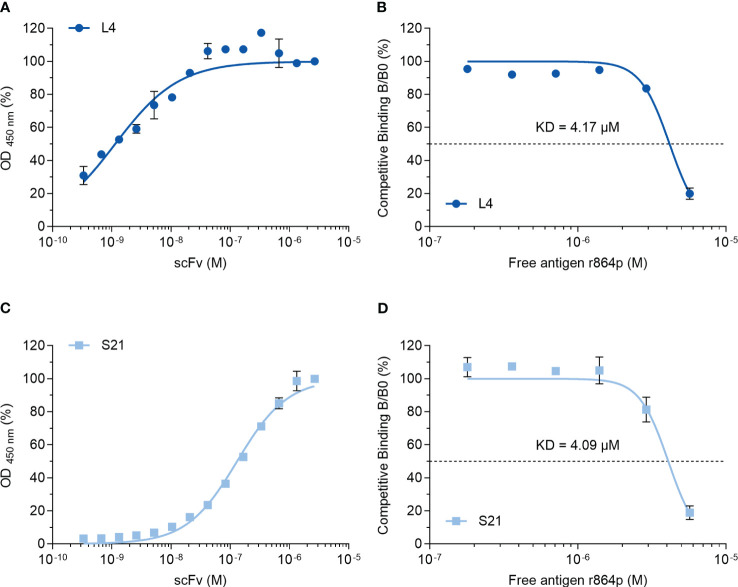
Binding curve and affinity determination of anti-nectin-4 scFvs. **(A)** Indirect ELISA showing the binding curve of L4 to nectin-4 ectodomain r864p fixed on plates. **(B)** Competitive ELISA of L4 to determine the dissociation constant (KD). Free r864p was serially diluted and incubated with L4 before the mixture was added to plates coated with r864p. **(C)** Indirect ELISA showing the binding curve of S21 to nectin-4 ectodomain r864p fixed on plates. **(D)** Competitive ELISA of S21 to determine the dissociation constant (KD). Free r864p was serially diluted and incubated with S21 before the mixture was added to plates coated with r864p. Mean ± SD are shown (N=2).

We next attempted to characterize the interaction between the scFvs and nectin-4. A binding curve analysis using non-competitive ELISA indicated that L4 binds to the recombinant nectin-4 fragment r864p efficiently, reaching 50% and 100% binding at 1.14 nM and 41.67 nM, respectively (**Figure 7A**). Subsequently, a competitive ELISA was conducted to determine the dissociation constant KD, which was approximately 4.17 μM. In contrast, a higher concentration of S21 was required to reach 50% binding (130.5 nM) and saturation in the non-competitive ELISA (**Figure 7B**), although the estimated KD (4.09 μM) is similar to that of L4.

**Figure 8:**
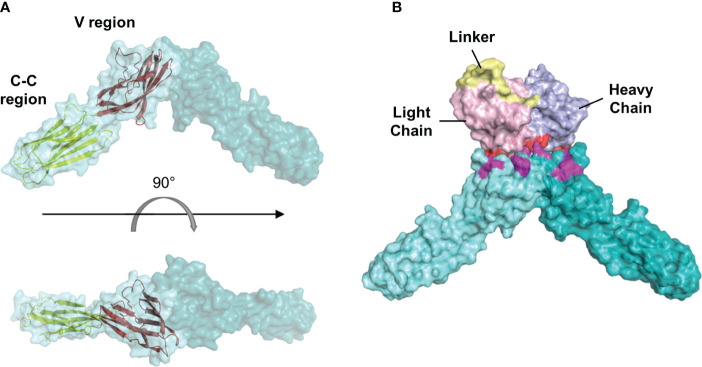
Molecular docking analysis of the interaction between scFv L4 and nectin-4. **(A)** Structure of human nectin-4 homodimer (4FRW) showing its one variable (V) and two constant (C-C) regions. **(B)** A representative docking frame showing the interaction between scFv L4 and nectin-4 homodimer structure. Polar contacts on the scFv (red) and nectin-4 (magenta) are shown.

To further predict possible binding sites between scFv and nectin-4, we selected L4 as an example to perform a protein-protein docking with the ectodomain of nectin-4. The sequence of scFv L4 is shown in **Supplementary Figure S2**. The homology model of L4 was generated and docked onto the nectin-4 homodimer structure (PDBID: 4FRW; **Figure 8A**). Our docking analysis indicated that L4 returned a probable binding frame on the tip of nectin-4 dimer and targeted the amino acids 57Asp, 58Ser, 85Lys, 88Leu, 100Gln, 101Pro, 105Arg, and 106Asn on nectin-4 (**Figure 8B**). This diversity in binding residues between L4 and nectin-4 potentially contributes to the efficient recognition of the soluble nectin-4 ectodomain by L4 as observed in the ELISA analyses (**Figure 7**).

## 4 Discussion

Multiple studies have suggested that nectin-4 may contribute to carcinogenesis. The extracellular portion of nectin-4 interacts with nectin-1 on the adjacent cell to promote cell-to-cell attachment, and it also interacts with integrin β4 on the same cell to activate the Src family kinases (SFKs) that sustain anchorage-independent growth of human mammary epithelial cells (18). Given the pleiotropic role of SFKs in cellular events, including cell cycle progression, cell survival, adhesion, and migration, and in pathophysiological disorders, including cancers (36), activation of SFKs by nectin-4 could contribute to cancer transformation from multiple pathways. The soluble nectin-4 ectodomain, which could be detected in the sera of breast cancer (37), lung cancer (9), and ovarian cancer (11, 38) patients, has been shown to interact with endothelial integrin β4 to promote angiogenesis in breast cancer through the Src-regulated PI3K/Akt pathway (19). This suggests that targeting or neutralizing the soluble nectin-4 in patient sera may be a potential therapeutic approach. In addition, nectin-4 is also considered a breast cancer stem cell marker, as its presence enhances cell invasion and epithelial-mesenchymal transition and activates the Wnt/β-catenin pathway through the PI3K/Akt axis (20). More recently, a study further identified nectin-4 as a cancer-specific ligand of the inhibitory receptor T-cell immunoreceptor with Ig and ITIM domains (TIGIT), and their interaction was found to inhibit the antitumor activity of nature killer (NK) cells (21). Consistent with these findings, clinical nectin-4 expression positively correlates with tumor size, histopathological grading, angiogenic markers, metastasis, and recurrence (22).

Given the importance of nectin-4 in tumor initiation and progression, antibodies against nectin-4 could be a helpful diagnostic/therapeutic tool. In the current study, we successfully generated nectin-4-targeted scFv libraries using the phage display technique (**Figures 1**, **2**). Selected clones L4 and S21 recognized the recombinant ectodomain of nectin-4 (**Figure 2B**) and successfully detected the endogenous nectin-4 in several breast cancer cell lines. Specifically, S21 demonstrated better performance in western blot and flow cytometry analyses (**Figure 3**), whereas L4 displayed high sensitivity and produced little background signal in immunofluorescence staining of 4% PFA-fixed cells (**Figure 4**) and IHC staining of paraffin-embedded breast cancer tissue sections (**Figure 5**). This could possibly be explained by molecular docking results indicating that L4 has a predicted binding site on the V loop junction of the nectin-4 dimer (**Figure 8**), which would only appear in its native conformation. S21, on the other hand, might recognize an epitope on the nectin-4 monomer that would be exposed upon cell dissociation. L4 is also more efficiently bound to fixed nectin-4 ectodomain in the non-competitive ELISA (**Figure 7**). These results suggest that S21 may be useful for laboratory applications such as flow cytometry and western blot detection, whereas L4 may be suitable for immunostaining, IHC, and potential development into a clinical diagnostic tool. Since the immunogens were based on the ectodomain, these scFvs could be utilized for both staining of the dissected tissues (**Figure 5**) and measuring the shed or soluble nectin-4 in patient sera and ascites, which can be indicative of disease status, therapeutic effect, and prognosis (9–11). In addition, since nectin-4 has been proposed as a new therapeutic target for antibody-based cancer treatment (39) and oncolytic measles virotherapy, which utilizes nectin-4 as a receptor (40–42), the scFvs could also be useful for screening suitable candidates to receive such nectin-4-targeted treatments.

Antibody-based therapeutics have been extensively studied in the past few decades, especially in the field of cancer treatment (43). Well-known examples include the HER2-directed mAbs, their derivatives conjugated with chemotherapeutic or immunotherapeutic drugs (44), and mAbs that target the vascular endothelial growth factor (VEGF) (45) for breast cancer treatment. Supporting the role of nectin-4 in cancer progression, it has been shown that blocking nectin-4 with antibodies could inhibit the growth of cell line-derived (18) and patient-derived (23) breast cancer mouse xenografts and augment the antitumor activity of NK cells (21). Importantly, our results also demonstrate that the scFvs can reduce the formation of attachment-free breast cancer cell aggregation (**Figure 6**), which can disrupt cell-cell contact and slow down tumor growth (18). Further analyses of the scFvs’ impact on tumor sphere formation and *in vivo* tumor suppression are underway. In addition, although the scFvs alone are not directly cytotoxic (**Supplementary Figure S1**), they can be explored through other strategies. For example, their anti-clustering effect could be useful in combination with cytotoxic anticancer agents to boost the anticancer effect. Moreover, conjugation with drugs or reporters is another popular strategy to increase the applicability of non-cytotoxic antibodies. For instance, in the therapeutic antibody-drug conjugate (ADC) enfortumab vedotin, the microtubule-disrupting agent monomethyl auristatin E (MMAE) was conjugated to the non-cytotoxic nectin-4-directed mAb AGS-22M6E to increase its tumor-killing effect. It was shown that enfortumab vedotin could inhibit breast, bladder, pancreatic, and lung cancer xenografts in mouse models (17) and has been further evaluated in multicenter phase 2 (EV-201; NCT03219333) and global phase 3 (EV-301; NCT03474107) trials, with preliminary results showing 44%-52% objective response rate (ORR) (46, 47) and prolonged survival compared to chemotherapy (48) in metastatic urothelial cancer patients who previously received platinum chemotherapy and anti-PD-1/PD-L1 immunotherapy. Based on the above observations, enfortumab vedotin has been granted accelerated approval by the U.S. Food and Drug Administration (FDA) for the treatment of metastatic urothelial cancer (39). It has also been shown that anti-nectin-4 antibody conjugated with the zirconium isotope ^89^Zr ([^89^Zr]AGS-22M6) could serve as a reagent for positron emission tomography (PET) evaluation of nectin-4-positive tumors and metastases *in vivo* (49). Nectin-4-targeting mAb conjugates ^99m^Tc-HYNIC-mAb_Nectin-4_ and mAb_Nectin-4_-ICG (Indocyanine green) were also developed for immuno-single photon emission computed tomography (SPECT) diagnostic imaging and photothermal therapy in TNBC-bearing mice (50). With a smaller size and faster clearance compared to intact mAbs, scFvs are highly suitable for the development of therapeutic or diagnostic purposes (2). Taking advantage of the better penetration of scFvs in target tumors (3), more scFv conjugates are being evaluated in clinical trials for cancer indications (51). Given that scFv L4 displayed high sensitivity and specificity in recognizing the native form of nectin-4 on breast cancer cell monolayer and tissue sections (**Figures 4**, **5**) and its ability to prevent nectin-4-positive tumor cell cluster formation (**Figure 6**), the scFv and its derivatives may be further developed and investigated for their diagnostic and therapeutic values. As scFv L4 was derived from chicken provenance, potential issues of immunogenicity and the scFv’s binding affinity to human nectin-4 could be further improved by humanization procedures (52).

Based on the protein-protein docking, we predicted multiple interacting residues on scFv L4 with the physiologically relevant nectin-4 homodimer (**Figure 8**). The amino acids’ diversity and the number of polar contacts that scFv L4 possesses in its interaction with nectin-4 homodimer (**Figure 8**) could, in theory, provide stability of the complex. In addition, the physical structure of L4 also has a non-occluded cleft between the light and heavy chains, which is broad and could potentially contribute to its binding affinity to nectin-4; whether this is because of the increased flexibility of the longer linker or because of the intra-sequence interactions warrants further analysis. Likewise, further in-depth examination of the quantitative binding energy and molecular dynamics simulation combined with biophysical analyses could also help better characterize L4’s complete protein binding profile.

In conclusion, we produced nectin-4-specific scFvs based on chicken IgY using the phage display method in this study. Two selected scFv clones could capture the ectodomain of nectin-4 and recognize endogenous nectin-4 on several breast cancer cell lines, with scFv L4 demonstrating better sensitivity and specificity to identify nectin-4 in its native form. Importantly, while the scFvs are non-cytotoxic, they could inhibit the self-clustering of nectin-4-positive breast cancer cells. Molecular docking analysis further revealed that the scFv L4 possibly binds to the tip of the nectin-4 homodimer junction. These results highlight the potential of developing the scFv clones for laboratory or clinical uses, either as a diagnostic tool or a therapeutic candidate for combination or drug conjugation to target nectin-4-positive cancers, including breast cancer.

## Data availability statement

The original contributions presented in the study are included in the article/**Supplementary Material**. Further inquiries can be directed to the corresponding authors.

## Ethics statement

The studies involving humans were approved by TMU-Joint Institutional Review Board. The studies were conducted in accordance with the local legislation and institutional requirements. The human samples used in this study were acquired from Taipei Medical University Joint Biobank. Written informed consent for participation was not required from the participants or the participants’ legal guardians/next of kin in accordance with the national legislation and institutional requirements. The experimental protocol for chicken immunization was approved by the Institutional Animal Care and Use Committee of Taipei Medical University.

## Author contributions

C-HLiu: Formal analysis, Investigation, Methodology, Writing – original draft, Writing – review & editing. S-JL: Conceptualization, Formal analysis, Funding acquisition, Methodology, Resources, Supervision, Writing – review & editing. C-HLee: Formal analysis, Investigation, Methodology, Writing – review & editing. C-YL: Formal analysis, Investigation, Writing – review & editing. W-CW: Formal analysis, Investigation, Writing – review & editing. B-YT: Resources, Writing – review & editing. Y-CL: Formal analysis, Writing – review & editing. C-LC: Methodology, Resources, Writing – review & editing. Y-YY: Conceptualization, Formal analysis, Funding acquisition, Methodology, Resources, Supervision, Writing – review & editing. L-TL: Conceptualization, Formal analysis, Funding acquisition, Methodology, Resources, Supervision, Writing – original draft, Writing – review & editing.
