# Supplementary material for: Production and characterization of single-chain variable fragment antibodies targeting the breast cancer tumor marker nectin-4
Source: Front Immunol. 2024 Jan 15;14:1292019. doi: 10.3389/fimmu.2023.1292019 (PMC10822971; doi:10.3389/fimmu.2023.1292019)
Supplement: Supplementary file 1 [file DataSheet_1.pdf]

Supplementary Material

Supplementary Table S1. Primers used for construction of scFv libraries.

**V_H with short linker**	
CSCVHo-F	5'-GGT CAG TCC TCT AGA TCT TCC GCC GTG ACG TTG GAC GAG -3'
CSCG-B	5'-CTG GCC GGC CTG GCC ACT AGT GGA GGA GAC GAT GAC TTC GGT CC -3'
**V_H with long linker**	
CSCVHo-FL	5'-GGT CAG TCC TCT AGA TCT TCC GGC GGT GGT GGC AGC TCC GGT GGT GGC GGT TCC GCC GTG ACG TTG GAC GAG -3'
CSCG-B	5'-CTG GCC GGC CTG GCC ACT AGT GGA GGA GAC GAT GAC TTC GGT CC -3'
**V_L with short & long linker**	
CSCVK-F	5'-GTGGCCCAGGCGGCCCTGACTCAGCCGTCCTCGGTGTC-3'
CKJo-B	5'-GGAAGATCTAGAGGACTGACCTAGGACGGTCAGG -3'
**Overlap extension**	
CSC-F	5'-GAG GAG GAG GAG GAG GAG GTG GCC CAG GCG GCC CTG ACT CAG -3'
CSC-B	5'-GAG GAG GAG GAG GAG GAG GAG CTG GCC GGC CTG GCC ACT AGT GGA GG-3'

Supplementary Figure S1. Cytotoxicity of the anti-nectin-4 scFvs were evaluated on breast cancer cells. Cells were seeded in 96-well plates (1 x 10^4 cells/well) and incubated with scFv L4 or S21 for 5 days. Cell viability (%) was assessed by Cell Counting Kit-8 (Sigma) following the manufacturer's instructions. Data shown are mean ± SD from three biological replicates.

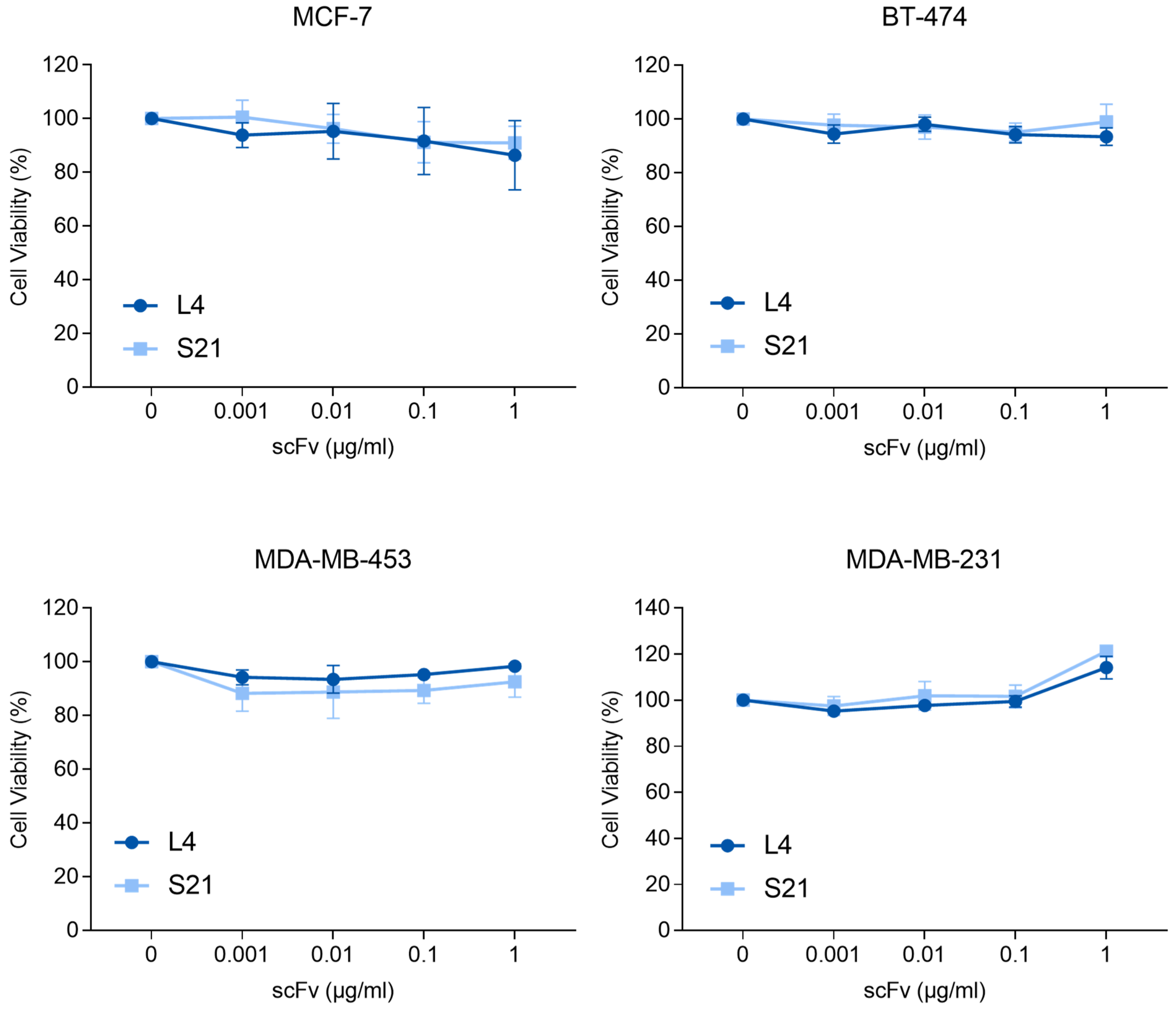

Supplementary Figure S2. Amino acid sequence of scFv L4.

ALTQPSSVSANPGETVKITCSGGSSNYYGWYQQKSPGSAPVTLIYNNNKRPSDIPS
RFSASKSGSTHTLTITGVRAEDEAVYFCGGWDKSAGIFGAGTTLTVLGQSSRSSGG
GGSSGGGGSAVTLDESGGGLQTPGGGLSLVCKASGFTFSSNGMAWVRQAPGKG
LEWVAGVNAAGSWTGYGAAVKGRATISRDNGQSTVRLQLNDLRAEDTGTYYCAKT
ADDWYGADDIDAWGHGTDVIVSS
